# Assessment of Liver Fibrosis with the Use of Elastography in Paediatric Patients with Diagnosed Cystic Fibrosis

**DOI:** 10.1155/2022/4798136

**Published:** 2022-03-19

**Authors:** Sabina Wiecek, Piotr Fabrowicz, Halina Wos, Bożena Kordys-Darmolinska, Maciej Cebula, Katarzyna Gruszczynska, Urszula Grzybowska-Chlebowczyk

**Affiliations:** ^1^Department of Paediatrics, Faculty of Medical Sciences, Medical University of Silesia, Katowice, Poland; ^2^Upper Silesian Child Health Centre, Katowice, Poland; ^3^University of Bielsko-Biała, Poland; ^4^Department of Radiodiagnostics and Invasive Radiology, Department of Radiology and Nuclear Medicine, School of Medicine in Katowice, Medical University of Silesia in Katowice, Poland; ^5^Department of Diagnostic Imaging, Department of Radiology and Nuclear Medicine, School of Medicine in Katowice, Medical University of Silesia in Katowice, Poland

## Abstract

**Background:**

Complications of cystic fibrosis-associated liver disease (CFLD) are a leading nonpulmonary cause of death. Noninvasive tests enabling early detection of liver changes, especially in children are sought. The aim of the study was to assess the scale of liver fibrosis with the use of elastography in paediatric patients with diagnosed cystic fibrosis (CF) and its comparison with other tests (APRI and Fibrotest).

**Methods:**

We examined 41 children, in the age range 2-21 years, with diagnosed CF. The analysis a included clinical picture, laboratory parameters of liver damage, and cholestasis. Aspartate aminotransferase-to-platelet ratio index (APRI) and Fibrotest were done in all patients. Liver stiffness measurements were acquired using shear-wave elastography (SWE).

**Results:**

CFLD was diagnosed in 16/41 patients (39%). Abnormal elastography was observed in 19/41 patients (46.3%), and in 5/41 (12.2%), the changes were advanced (F4). Abnormal elastography was observed in 12/16 (75%) of the patients with CFLD, and in 7/25 (28%), there were no lesions observed in the liver in the course of cystic fibrosis. In all patients with F4, we observed abnormal results of the APRI and Fibrotest. In most patients with small changes in elastography, we found normal results of the APRI and Fibrotest.

**Conclusion:**

Elastography seems to be a noninvasive examination useful in everyday clinical work in detecting early liver changes and monitoring of progression in paediatric patients with diagnosed cystic fibrosis, ahead of changes in laboratory tests. The cost-effectiveness of this test, the possibility of its repetition, and its availability are additional benefits.

## 1. Introduction

Cystic fibrosis (CF) is a genetic, multiorgan, and chronic disorder affecting mainly the respiratory and gastrointestinal systems, including the liver and the pancreas. The gastrointestinal symptoms are related to pancreatic exocrine insufficiency which disturbs the processes of digestion and absorption, which causes chronic steatorrhea, stomach pain, and the resulting insufficient body mass and height. Changes within the liver and bile ducts are less frequently observed [1–3]. Even though liver abnormalities concern only 5-20% of patients with cystic fibrosis, they shorten the lifespan and deteriorate its quality. Liver-related morbidities are the most common extrapulmonary cause of death in patients with CF. Changes in the liver in the course of cystic fibrosis are a network of complex processes of fibrosis, inflammation, remodelling, apoptosis, and cholestasis resulting from the abnormal functioning of the CFTR protein, immunological reactions, and response to oxidative stress. The most common changes observed in the liver and bile ducts involve focal fibrosis, liver steatosis, biliary cirrhosis, portal hypertension, and/or choledocholithiasis. In the majority of patients with cystic fibrosis, the course of liver complications is initially asymptomatic. Sometimes, itchiness of the skin and jaundice develop but mainly in patients with more advanced courses of the disease. The first symptom is usually hepatosplenomegaly and/or abnormal results of the laboratory tests. It is believed that liver test results elevated above the norm in at least 2 parameters over at least 3 months indicate progressing changes in the organ. However, laboratory tests have relatively low sensitivity and specificity. The majority of patients with multifocal cirrhosis have normal results of the laboratory tests. An isolated increase in the activities of aminotransferases with normal values of GGTP may indicate liver steatosis [4–12]. The Doppler ultrasonography may show steatosis, features of portal hypertension, and cirrhotic changes in the liver. This test is cost-effective and noninvasive; however, a normal-looking liver USG does not exclude the ongoing process of fibrosis. Liver biopsy with the histopathological assessment of fibrosis is an invasive and painful procedure and is prone to complications and sampling error (not evenly distributed steatosis and fibrosis). The biopsy sample accounts for only 1/50000 of the total liver mass and therefore does not reflect the function or the degree of damage of the organ. Hence, noninvasive tests which would allow the detection of early changes in the liver and bile ducts in the course of cystic fibrosis are sought. Dynamic elastography is a method of noninvasive, quantifying the assessment of the hardness of liver parenchyma (and therefore indirectly of fibrosis) by measuring the speed of acoustic dispersion in it. It is a physical way of assessing the speed of a low-frequency (50 Hz) acoustic wave and energy passing through liver parenchyma. The speed of the wave is directly proportional to the stiffness and thus indirectly to the degree of liver fibrosis (kPa). The results of studies conducted by many authors are encouraging and show a good correlation between the parameters of liver fibrosis and steatosis assessed with elastography and biopsy in children with chronic liver diseases [13–18]. There are few reports in the subject literature on the usefulness of this examination for the early detection of changes in the liver in the course of cystic fibrosis in paediatric patients.

### 1.1. Objective

The aim of the study was to assess the degree of liver fibrosis using elastography in paediatric patients with diagnosed cystic fibrosis and its comparison with other tests measuring liver fibrosis (APRI and Fibrotest).

### 1.2. Methods

We examined 41 patients with diagnosed cystic fibrosis, aged from 2 years to 21 years (the average age of 9.6 years), 24 girls (58.5%) and 17 boys (41.5%), diagnosed and treated in the Department of Paediatrics of the Medical University of Silesia in Katowice.

### 1.3. Inclusion Criteria

The inclusion criteria involved the diagnosis of CF confirmed by genetic testing and being aged 2 to 21 years. Each patient or their legal guardian signed an informed consent form to participate in the study.

### 1.4. Exclusion Criteria

Patients aged less than 2 years and/or with accompanying other liver diseases were excluded.

The analysis included age, sex, clinical symptoms, laboratory tests for exocrine and endocrine pancreatic efficiency (elastase activity and steatocrit in the stool, glucose concentration in the blood), laboratory parameters of liver damage, and cholestasis (activity of alanine and aspartate aminotransferases, alkaline phosphatase, gamma glutamyl-transpeptidase, concentration of albumin, bilirubin, and INR). Fibrotest and APRI were calculated in all patients.

The studied group of patients was divided into 2 subgroups: one cohort with the Debray criteria for CFLD, and the other one, without. The Debray criteria include abnormalities in the physical examination such as hepato-/splenomegaly, abnormal test results indicating damage to the liver and cholestasis, and abnormal ultrasound [8, 9]. Subgroup 1 consisted of 16 children (39%) with diagnosed CFLD, 7 boys (43.75%) and 9 girls (56.25%). The patients were aged 2-18 with an average age of 9.31. Subgroup 2 consisted of 25 patients (61%) without diagnosed CFLD (10 boys (40%) and 15 girls (60%), aged 2.5-21 years with an average age of 9.88).

APRI was calculated using the formula of Wai et al. [19]: [AST/upper limit of normal (ULN)/platelet count (expressed as platelets × 10^9^/L) × 100].

Fibrotest was calculated from the logarithmic equation taking the following parameters into consideration: alpha2-macroglobulin, A1 apolipoprotein, haptoglobin, the concentration of bilirubin, the activity of GTP and of aspartate aminotransferase, age, and sex. The values of <0.25 were considered normal.

The APRI and Fibrotest values were calculated in patients, following the period of at least eight hours fasting, with no indicators of an acute infection.

The elastography and ultrasonography examinations of the abdomen with the Doppler option were performed in all patients with diagnosed cystic fibrosis. All examinations were performed using S-Shearwave™, which is a point shear-wave method, with Samsung RS85 ultrasound machine with CA1-7A convex probe (S-SWE; Samsung Medison Co., Ltd., Seoul, Korea) by one board-certified radiologist, with 2 years of experience in elastography and 11 years in paediatric abdominal ultrasonography. Ten measurements were taken in 3 different sites in the hepatic right lobe through an intercostal space, in accordance with the European Federation of Societies for Ultrasound in Medicine and Biology (EFSUMB) updated guidelines [20] and Samsung recommendations [21]. Results were expressed in kilopascal (kPa) (Table 1).

The study protocol was approved by the Bioethics Committee of the Silesian Medical University in Katowice (KNW/0022/KBI/147/17/18).

### 1.5. Statistical Analysis

The statistical analysis was performed using the procedures available in the MedCalc v17.7 licensed software. The quantitative variables were presented as an arithmetic mean and the standard deviation (normally distributed variables) or a median and the interquartile range (variables of not normal/skewed distribution). The normality of distribution was assessed with the Shapiro-Wilk test. Qualitative variables were presented as absolute values and percentages. The intergroup differences for the quantitative variables were assessed with an analysis of variance (normally distributed variables) or the Kruskal-Wallis test (variables of skewed distribution). In the case of statistically significant differences within many groups revealed by the ANOVA or Kruskal-Wallis tests, a post hoc-type analysis was performed. Fisher's exact test or chi-square test was performed for qualitative variables. Relationships between quantitative variables were assessed with Spearman's rank correlation coefficient. Statistical significance was established at *p* < 0.05.

## 2. Results

The following symptoms dominated the clinical picture of the studied patients with diagnosed cystic fibrosis: symptoms from the respiratory system (38/41-92.7%), pancreatic insufficiency (36/41-87.8%), and nutritional disorders (14/41-34.1%). Features of liver dysfunction (CFLD) assessed with the Debray criteria were observed in 16/41 (39.02%) of the analysed patients. The delF508 mutation was reported most frequently -93.7% (homozygosity: 23/41-56.1%, heterozygosity: 15/41-36.6%). The delF508 mutation was also observed more often in patients with diagnosed CFLD; however, they were not statistically significantly different. In the group of patients with CFLD, malnutrition (43.75% vs. 28%) was reported more frequently, while pancreatic insufficiency (81.25% vs. 92%) and symptoms from the respiratory tract (87.5% vs. 96%) were less. However, the differences were not statistically significant. No correlation between the presence of changes in the liver (CFLD) and meconium ileus and the history of the salt-losing syndrome was reported. In the patients with confirmed abnormal elastography, malnutrition was reported statistically significantly more often.

The clinical picture of the studied patients with diagnosed cystic fibrosis is shown in Table 2.

Normal elastography (F0-F1) was reported in 22/41 (53.7%) patients and advanced changes, F3-F4 in 5/41 (12.2%) Table 3.

Elevated activities of aminotransferases were observed in 14/41 (34.1%) of the patients and elevated parameters of cholestasis in 10/41 (24.4%) of the patients. Abnormal APRI was observed in 7/41 (17.1%), and Fibrotest in 10/41 (24.4%).

The average values for APRI and elastography were statistically significantly higher in patients with coexisting CFLD (average values of APRI 0.37 vs. 0.14, average values for elastography 7.73 kPa vs. 3.62). Increased values of APRI only concerned patients with CFLD; none of the patients without coexisting CFLD had abnormal APRI values. Abnormal elastography was reported in 75% of patients with CFLD and in 25% of those without diagnosed CFLD—those changes corresponded with F2. Changes corresponding with advanced fibrosis F3 were shown in only 2/41 (4.9%), and with cirrhosis (F4) in 3/41 (7.3%)—they were patients with the CFLD diagnosed based on the Debray criteria. Average values for Fibrotest were higher in patients with CFLD than in those without (0.145 vs. 0.1); however, they were not statistically significantly different. Abnormal results of the Fibrotest were observed in half of the patients with CFLD and only in 8% (2 children) of the patients with the normal functions of the liver in the course of cystic fibrosis (Table 4).

The abdominal ultrasound showed abnormalities in 16/41 (39%), mainly in the form of steatosis (13/41-31.7%) and hepatomegaly (8/41-19.5%). Features of periportal fibrosis were reported in 5/41 (12.2%) of patients with diagnosed cystic fibrosis, and splenomegaly in 6/41 (14.6%). In 2 patients, the Doppler flow abnormalities in the form of portosystemic shunt were observed.

No correlation between the results of elastography and the age, gender, type of mutation, pancreatic insufficiency, and the symptoms from the respiratory tract was shown. However, there was a link between insufficient body mass and the result of elastography. Children with advanced changes in elastography (F3 and F4) had lower BMI, and this was a statistically significant difference.

There was also a connection between the advancement of the changes revealed in elastography and the results of the APRI and Fibrotest. Abnormal APRI was observed in all the patients with an abnormal result of elastography of F3 and F4. Abnormal APRI was also noted in two patients with the elastography of F2. However, in the majority of patients with the F2 level of advancement, the result of APRI was normal. All patients with the normal elastography had normal values of APRI.

In all patients with the changes in elastography of F3-F4, abnormal results of the Fibrotest were concluded. In the majority of patients with the elastography of F2, normal values of APRI (12/14-85.7%) and of the Fibrotest (9/14-64.3%) were observed (Table 5.)

## 3. Discussion

Lesions within the liver affect a high proportion of patients with diagnosed cystic fibrosis and may lead to fibrosis, cirrhosis, and/or portal hypertension. They most often begin in infancy and/or the postneonatal period. From a clinical point of view, early detection of such lesions will contribute to the inhibition/regression of the pathological process, preventing the development of complications. However, changes in the liver in patients with diagnosed cystic fibrosis are usually asymptomatic or show few symptoms and are suspected when the conducted ultrasound shows elevated parameters of liver damage and/or cholestasis and hepatosplenomegaly. At this stage, such changes are already advanced and irreversible. Therefore, the search for the diagnostic markers which would allow for early detection of changes in the liver in the course of cystic fibrosis, and simultaneously facilitating the assessment of the already ongoing changes seems important [6, 8, 9, 22–24]. Among our patients, the features of abnormal liver functions in the course of cystic fibrosis (CFLD) as per the Debray criteria were observed in 16/41 people (39%), and in the majority of cases, they were detected during routine laboratory tests and scans (abdominal ultrasound). Friedrich-Rust et al. analysed the clinical picture of cystic fibrosis in 106 patients, both paediatric and adult, and observed the features consistent with CFLD in 22.6% [25]. Chryssostalis et al. reported the presence of lesions in the liver in 90 out of 285 adult patients (32%) over the age of 18 (the average age of 34.5) with diagnosed cystic fibrosis, and liver cirrhosis in 23/285 (8%) [5]. This further highlights the importance of this clinical problem in patients with diagnosed cystic fibrosis.

As for our patients with changes in the liver (CFLD), the delF508/delF508 mutation was the dominating one, and in the clinical picture of the majority of patients, pancreatic insufficiency coexisted with symptoms from the respiratory tract. Ciuca et al. showed a more frequent occurrence of changes in the liver in patients aged 18 and below and with the delta F508/deltaF508 mutation, and also with the meconium ileus in the anamnesis [1]. Similarly, Lam et al. reported statistically significantly more frequent pancreatic insufficiency in the paediatric population with CFLD (79% vs. 63%) [17]. Stonebraker et al. analysed 561 of patients with cystic fibrosis, aged 2 to 52, and 99% had pancreatic insufficiency and 92% deltaF508 mutation. According to their observations, the average age of the onset of CFLD was 10. In the case of our patients, this number was lower, 6 years of age [26]. Hence, it is important to conduct CFLD studies in the paediatric population. From a clinical point of view, gene therapies may, in the future, prevent the development of liver lesions.

Elastography is a noninvasive method to measure liver stiffness. Several authors delineate a role for transient elastography (TE) in the diagnosis of advanced CFLD with portal hypertension and its superiority over other diagnostic methods, including grey-scale ultrasound. Transient elastography is recognized as a reliable tool to diagnose cirrhosis and distinguish healthy from fibrotic liver tissue, but it is less accurate for the differentiation between mild and moderate fibrosis. Nevertheless, studies in adults have shown that TE is a useful tool for monitoring disease progression [18, 27–30].

Van Biervliet et al. analysed 150 patients with diagnosed cystic fibrosis, aged 9-24. They concluded CFLD in 20 (14%) patients following the first elastography and 5 developed CFLD within the 6 years of observation. The average values of elastography were 14 kPa (8.7-32.2) compared with the 5.3 (4.9-5.7) of cystic fibrosis without CFLD [14]. In their study, Klotter et al. measured the suitability of liver stiffness elastography in the progression of changes in the liver in cystic fibrosis over the course of a 5-year observation. They established the value of 6.3 kPa as the marking line for advanced changes [16].

As concerns our patients, we also reported statistically significant differences in the results of elastography between the group with diagnosed CFLD and without (7.73 vs. 3.62); however, the values were lower than in the studies conducted by Van Biervliet et al. and Klotter et al. This may be due to our study group being younger and having less advanced changes in the liver.

Kitson et al. have shown that elastography > 6.8 kPa predicts CFLD with 76% sensitivity, 84% specificity, and a positive likelihood ratio of 9.5 [30].

Malbrunot-Wagner et al. measured the correlation between elastography and endoscopic changes in 18 patients, aged less than 18 with diagnosed portal hypertension in the course of cystic fibrosis. In the patients with diagnosed oesophageal varices, the results of elastography were on average 22.4 kPa (14.4-30.4) compared with the patients without such varices, 7.9 kPa (4.4-13.7) [31].

Lam et al. believe that the stiffness elastography of over 8.9-12 kPa largely correlates with the presence of oesophageal varices [17]. We also observed the presence of oesophageal varices in our patients with the elastography score of F4 (>9.7 kPa) changes in elastography.

### 3.1. Elastography and Age, Sex, and Clinical Symptoms

Friedrich-Rust and Menten et al. reported higher elastography results in men compared with women (4.6 vs. 3.9 kPa); however, they did not show a link with age [18, 25]. Our observations did not confirm that. We observed that the values of elastography increased with age; however, they were not statistically significant differences. Similarly, Lewindon et al. and Goldschmidt et al. did not confirm a correlation between the result of elastography and patients' age [15, 32]. Gominon et al. in their study have shown that liver stiffness increases with time in patients with CF and that the slope of worsening is greater in patients who will develop CFLD. They have observed an average 6% progression in liver stiffness per year, in a population of paediatric patients with CF [11]. Similar results have been found by Karlas et al.; they have shown a 9% increase in liver stiffness during a period of 22.5 months in 41 adults patients with CF [33]. In our study, we plan to perform elastography every 6 months, which we hope will become the norm in everyday clinical practice.

In our study, we observed a link between the result of elastography and BMI—the lower the BMI, and therefore the level of undernourishment, the higher the stiffness values shown in elastography. However, Gominon et al. did not report such a link in their study of paediatric patients with diagnosed CFLD [11]. It was concluded that children with diagnosed cystic fibrosis and coexisting liver damage have lower body mass, lower height, and lower arm circumference and BMI. This group is also reported to have a significantly low level of linoleic (LA), docosahexaenoic (DHA), and docosapentaenoic (DPA) acids [6, 24].

### 3.2. Elastography and APRI and Fibrotest

Early detection of fibrotic lesions in the liver seems to be particularly difficult. Minimally invasive tests characterised by high sensitivity and specificity are therefore the subject of interest. Among laboratory tests assessing the processes of liver fibrosis in the course of cystic fibrosis, the most widely available is APRI and Fibrotest. APRI is an indirect biochemical marker of hepatic fibrosis. In our patients with diagnosed cystic fibrosis, we reported abnormal values of APRI in 7/41 (17.1%). All patients with the elastography of F3-F4 had an abnormal value of APRI (5/41). Two children with an abnormal value of APRI in elastography had F2-type changes, which indicates that they are less advanced. Also Kitson et al. in their study compared the link between APRI and the results of elastography and showed high correspondence between these two tests in advanced changes, and lower sensitivity and specificity at an early stage [30].

Calvopina et al. tested 125 children with confirmed cystic fibrosis (CFLD changes in 55/125, no changes in the liver in 41/125) and 29 belonging to the control group. The average results of elastography were 8.1 kPa, 6.2 kPa, and 5.3 kPa, respectively. The combination of elastography and APRI proved to have 14.8 times higher sensitivity in the detection of CFLD (AUC = 0.84) compared with single tests [34]. Similar conclusions were reached by Alexopoulou et al., Aqul et al., and Koh et al. [27, 35, 36].

Patients with splenomegaly, low count of platelets and oesophageal varices had the values of liver stiffness in elastography of >20 kPa. The result of APRI closely correlated with the result of elastography in this group of patients. These results were confirmed in the studies by Sadler et al. and our observations [37].

Klotter et al. observed that both APRI and Fibrotest closely correlated with the results of elastography and the advancement of changes in the liver in the course of cystic fibrosis. In their study, they analysed 60 patients (16 adults and 44 children) with cystic fibrosis. They has calculated a cut-off for the rise in liver stiffness of >0.38 kPa/year to be optimal for the identification of children with progressively increasing liver stiffness [16]. Van Biervliet et al. concluded that all their patients with portal hypertension had abnormal levels of elastography, of F3-F4 and of Fibrotest [14].

Sadler et al. analysed the efficacy of noninvasive tests, including APRI, Fibrotest, and elastography in 127 adult patients with confirmed cystic fibrosis in the detection of changes in the liver. The results of elastography, APRI, and Fibrotest were statistically significantly higher in patients with CFLD than in those without. Having compared the above-mentioned results with those conducted in our patients, we also noticed statistically significant differences between the patients with and without CFLD in the results of APRI (0.37 vs. 0.14) and elastography (0.73 vs. 3.62). The results of the Fibrotest were higher in the patients with CFLD (0.145 vs. 0.100), but they were not statistically significant [37].

Karlas et al. measured the efficacy of noninvasive tests involving Fibrotest, elastography, and APRI in 55 adults with confirmed cystic fibrosis. They observed significant differences in the elastography depending on the advancement of the changes in the liver: cirrhosis/no signs of cirrhosis CFLD (7.95 vs. 4.16 kPa), *p* < 0.005. They believe that APRI and Fibrotest were the best tests for the detection of the early changes, and the results of elastography correlated with those of APRI (Rho > 0.4) [33].

According to Pavlov et al., elastography can fully replace liver biopsy with histopathology in the case of advanced changes within the liver (>F2). Unfortunately, as they also reported, elastography had a much lower sensitivity and specificity when the changes in the liver were less advanced [38].

Additionally, Van der Feen et al. showed that elastography may be used to monitor the treatment with ursodeoxycholic acid in the course of cystic fibrosis. They analysed 105 patients with confirmed cystic fibrosis. In the patients without the treatment with UDCA, the liver stiffness increased by 0.19 kPa over a year and decreased by 0.70 kPa over a year in the patients who received UDCA, which confirms the efficacy of the supplementation with UDCA in the prevention/reduction of liver fibrosis in the course of cystic fibrosis [39].

The downside of the study is the fact that the groups of patients are not very numerous. Nevertheless, cystic fibrosis is considered a “rare disease” affecting around 1/5000 people in Europe. It would certainly be worth comparing the results of elastography with the assessment of liver fibrosis in the histopathology of the organ. However, liver biopsy is an invasive and not risk-free procedure which only shows topical fibrotic and inflammatory changes, and not the holistic picture.

On the other hand, there are very few studies that assess the suitability of minimally invasive procedures for the detection of early lesions in the liver in paediatric patients with cystic fibrosis. And it is children below the age of 10 that are most frequently diagnosed with CFLD. We are planning on repeating the elastography in this group of patients after 5 and 10 years to assess the progression of fibrosis in paediatric patients.

## 4. Conclusions

Elastography seems to be a noninvasive examination useful in everyday clinical work in detecting early liver changes and monitoring of progression in paediatric patients with diagnosed cystic fibrosis, ahead of changes in laboratory tests. The cost-effectiveness of this test, the possibility of its repetition, and its availability are additional benefits.

In this study, we demonstrate the usefulness of combining transient elastography with APRI and Fibrotest to both confirm the presence of CFLD and differentiate early-moderate fibrosis from advanced severe fibrosis of the liver in paediatric patients with cystic fibrosis. The detection of early lesions may in the future lead to earlier treatment and prevent the progression of the disease.

## Figures and Tables

**Table 1:** Lesion scale assessment in liver elastography.

Stages of liver fibrosis	METAVIR score	Stiffness (kPa)
No fibrosis or portal fibrosis without septa	F0-F1	<3.95
Portal fibrosis with a few septa	F2	≤3.95, <7.0
Septal fibrosis with many septa but no cirrhosis	F3	≤7.0, <9.7
Cirrhosis	F4	≥9.7

**Table 2:** The clinical picture of the examined patients with diagnosed cystic fibrosis.

	Values of total examined group *N* = 41	Patients with CFLD (subgroup 1) *n* = 16 (39%)	Patients without CFLD (subgroup 2) *n* = 25 (61%)	Normal elastography F0-F1 *N* = 22 (53.7%)	Abnormal elastography F2-F4 *N* = 19 (46.3%)
Age (years)	2.0-21.0 (mean: 9.6)	2.00-18 (mean: 9.31)	2.5-21.0 (mean: 9.88)	2.5-21.0 (mean: 9.42)	2.0-18.0 (mean: 9.74)
	NS			NS	
Sex: female/male (*n*/*n*)	24 (58.5%)/17 (41.5%)	9 (56.25%)/7 (43.75%)	15 (60%)/10 (40%)	14 (63.3%)/8 (36.7%)	10 (45.5%)/9 (54.5%)
	NS			NS	
Mutation (*n* (%))					
delF508/delF508	23/41 (56.1%)	11/16 (68.75%)	12/25 (48%)	10/22 (45.5%)	13/19 (68.4%)
delF508/other	15/41 (36.6%)	4/16 (25%)	11/25 (44%)	10/22 (45.5%)	5/19 (26.3%)
Other	3/41 (7.3%)	1/16 (6.25%)	2/25 (8%)	2/22 (9%)	1/19 (5.3%)
	NS			NS	
Shwachman-Kulczycki score (pts)	74.81 (range 50-110)	72.36 (range 50-105)	78.21 (range 55-110)	77.33 (55-110)	75.42 (50-105)
	NS			NS	
Clinical features (*n* (%))					
Pancreatic insufficiency	36/41 (87.8%)	13/16 (81.25%)	23/25 (92%)	20/22 (90.0%)	16/19 (84.2%)
	NS			NS	
Symptoms from the respiratory tract/recurrent respiratory tract infection	38/41 (92.7%)	14/16 (87.5%)	24/25 (96%)	20/22 (90%)	18/19 (94.7%)
	NS			NS	
Malnutrition (body mass index < 3 pcn, taking into account age, sex, and population)	14/41 (34.1%)	7/16 (43.75%)	7/25 (28%)	5/22 (22.7%)	9/19 (47.4%)
	NS			*p* < 0.05	
Liver dysfunction	16/41 (39.0%)	16/41 (39.0%)	0/25 (0%)	0/22 (0%)	16/19 (84.2%)
Salt loss syndrome	7/41 (17.1%)	3/16 (18.75%)	4/25 (16%)	4/22 (18.2%)	3/19 (15.8%)
	NS			NS	
History of meconium ileus/treated surgically	6/41 (14.6%)	2/16 (12.5%)	4/25 (16%)	4/22 (18.2%)	2/19 (10.5%)
	NS			NS	

**Table 3:** Results of elastography in the patients with diagnosed cystic fibrosis.

Result of elastography	F0-F1	F2	F3	F4
Number of patients	22/41 (53.7%)	14/41 (34.1%)	2/41 (4.9%)	3/41 (7.3%)

**Table 4:** Parameters of liver function and fibrosis in the examined patients with CF.

Parameters	Total examined group *N* = 41			Patients with CFLD *N* = 16			Patients without CFLD *N* = 25		
	Range of activity	Mean activity	Number of patients with abnormality	Range of activity	Mean activity	Number of patients with abnormality	Range of activity	Mean activity	Number of patients with abnormality
APRI	0.1-2.1	0.28	*7/41 (17.1%)*	*0.1-2.1*	*0.37*	*7/16 (43.75%)*	*0.1-0.3*	*0.14*	*0/25 (0%)*
				p < 0.005					
Fibrotest	0.04-0.30	0.14	*10/41 (24.4%)*	*0.04-0.30*	*0.145*	*8/16*	*0.04-0.29*	*0.1*	*2/25 (8%)*
				p = 0.278					
Elastography (kPa)	2.8-35.6	5.42	*19/41 (46.3%)*	*3.2-35.6*	*7.73*	*12/16 (75%)*	*2.8-4.3*	*3.62*	*7/25 (28%)*
				p < 0.005					

**Table 5:** The comparison of the results of elastography and the APRI indicator and of the Fibrotest and Actitest in the patients with diagnosed cystic fibrosis.

Results of elastography	F1	F2	F3	F4	Total	
APRI						
Normal	*22/41 (53.7%)*	*12/41 (29.2%)*	*0/41 (0.0%)*	*0/41 (0.0%)*	*34/41 (82.9%)*	p = 0.0009
Abnormal	*0/41 (0%)*	*2/41 (4.9%)*	*2/41 (4.9%)*	*3/41 (7.3%)*	*7/41 (17.1%)*	
Total	*22/41 (53.7%)*	*14/41 (34.1%)*	*2/41 (4.9%)*	*3/41 (7.3%)*	*41/41 (100%)*	
Fibrotest						
Normal	*22/41 (53.7%)*	*9/41 (21.9%)*	*0/41 (0.0%)*	*0/41 (0.0%)*	*31/41 (75.6%)*	p = 0.043
Abnormal	*0/41 (0.0%)*	*5/41 (12.2%)*	*2/41 (4.9%)*	*3/41 (7.3%)*	*10/41 (24.4%)*	
Total	*22/41 (53.7%)*	*14/41 (34.1%)*	*2/41 (4.9%)*	*3/41 (7.3%)*	*41/41 (100%)*	

## Data Availability

Data and results are stored and accessed in Department of Paediatrics Silesian Medical University in Katowice.

## Abbreviations

CFLD: Cystic fibrosis-associated liver disease
CF: Cystic fibrosis
CFTR: Cystic fibrosis transmembrane conductance regulator
ALAT: Alanine aminotransferase
AspAT: Aspartate aminotransferase
GGTP: Gamma glutamyl-transpeptidase
AF: Alkaline phosphatase
APRI: Aspartate aminotransferase-to-platelet ratio index
SWE: Shear-wave elastography
INR: International normalized ratio
TE: Transient elastography
